# One-year metreleptin in Colombian sisters with congenital leptin deficiency

**DOI:** 10.1080/21623945.2025.2508188

**Published:** 2025-05-26

**Authors:** Hernan Yupanqui-Lozno, Jancy Andrea Huertas-Quintero, Maria E. Yupanqui-Velazco, Rocío A. Salinas-Osornio, Carlos M. Restrepo, Adriana Gonzalez, Edna J. Nava-Gonzalez, Luis G. Celis-Regalado, Constanza Neri Morales, Victor M. Hernandez-Escalante, Julio Licinio, Hugo A. Laviada-Molina, Ernesto Rodriguez-Ayala, Carlos Arango, Raul A. Bastarrachea

**Affiliations:** a Clinical Research Department, Dexa Diab IPS, Bogotá, Colombia; b Hospital Universitario Mayor, Universidad del Rosario, Bogotá, Colombia; c Centro de Investigación y Desarrollo Científico, Ciencias de la Salud, Universidad del Valle de Atemajac (UNIVA), Zapopan, México; d GeniURos, CIGGUR, Instituto de Medicina Traslacional, Escuela de Medicina y Ciencias de la Salud, Universidad del Rosario, Bogotá, Colombia; e Facultad de Salud Pública y Nutrición (Faspyn), Universidad Autónoma de Nuevo León, Monterrey, México; f Facultad de Medicina, Universidad de La Sabana, Chía, Colombia; g Facultad de Medicina, Universidad Autónoma de Yucatán, Mérida, México; h Department of Genetics, SUNY Upstate Medical University, Syracuse, NY, USA; i Escuela de Ciencias de la Salud, Universidad Marista de Mérida, Yucatan, México; j Population Health Program, Texas Biomedical Research Institute, San Antonio, TX, USA

**Keywords:** Congenital leptin deficiency, Colombian sisters, *LEP* gene, recombinant human leptin (metreleptin); leptin replacement therapy

## Abstract

We discovered two adult sisters in Colombia, lineally consanguineous, with severe obesity and undetectable serum leptin levels despite markedly elevated body fat. Their clinical profile included childhood-onset extreme weight gain, intense hunger, hyperphagia, hypogonadotropic hypogonadism, and family history of obesity. Direct sequencing of the LEP gene revealed a novel homozygous missense mutation in exon 3 (c.350G>T [p.C117F]). The presence of this mutation, undetectable leptin, and severe obesity confirmed a diagnosis of monogenic leptin deficiency. Here we describe the clinical outcomes of a 12-month treatment with recombinant human leptin (metreleptin). Metabolic and endocrine assessments were conducted before and after therapy. Metreleptin therapy significantly reduced BMI: from 59 to 38 kg/m^2^ (OBX1, age 27) and 60 to 48 kg/m^2^ (OBX2, age 24). Total body fat mass decreased, serum lipids normalized, and insulin sensitivity improved. Hypogonadotropic hypogonadism reversed, and menstruation resumed. Thus, metreleptin reversed the major metabolic and endocrine abnormalities associated with leptin deficiency in these sisters. Limitations include the small sample size, absence of a control group, and lack of anti-metreleptin antibody measurements. Nevertheless, our findings support that leptin replacement with metreleptin is currently the only effective hormonal treatment for this monogenic form of human obesity.

## Introduction

The cloning of leptin in 1994 was an important milestone in obesity research. It led to outstanding advances in understanding the regulation of energy balance in rodents and humans, leading to a new era in our knowledge of adipose tissue metabolism and feeding behaviour [1,2]. Leptin (from the Greek word leptos, which means thin) is a key regulator of neuroendocrine function and energy homoeostasis [3,4]. Leptin is mainly secreted by adipose tissue and its circulating blood levels reflect whole-body energy stores proportional to total fat mass and energy deprivation [5]. Leptin acts directly in the hypothalamus through activating the leptin receptor (ObRb) [6]. Leptin activates pro-opiomelanocortin (POMC) – containing neurons, which produce anorexigenic molecules and deactivates the orexigenic neuropeptide Y (NPY) – and agouti-related peptide (AgRP) – containing neurons. With low body fat levels or during fasting, leptin levels decrease, POMC neuronal activity decreases, and NPY and AgRP neural activity increases, resulting in increased appetite and food intake. The opposite occurs when body fat stores are abundant driving leptin’s actions to decreased food intake [7,8].

It has been suggested that the primary role of leptin is to allow overnutrition and deposition of body fat without metabolic injury to non-adipose tissues [9,10]. The prevalence of genetic obesity is estimated to be approximately 7% among patients with early-onset severe obesity. However, in certain ethnic groups with higher rates of consanguinity, this prevalence is believed to be significantly higher. To date, 165 patients with variants in LEP and LEPR have been reported in the literature. Additionally, separate studies have reviewed the clinical and molecular characteristics of variants in LEP and LEPR [11]. Leptin deficiency can result from congenital generalized lipodystrophy (CGL) [12] and congenital leptin deficiency (CLD) [13], in which leptin’s biological actions are absent because of a mutation in the leptin gene. In these conditions, plasma leptin levels are negligible and cannot rise in response to a caloric surplus. Such patients exhibit early onset of severe and generalized steatosis and hyperlipidaemia. Severe type 2 diabetes owing to β-cell damage, profound insulin resistance, and marked hepatic steatosis resulting from leptin deficiency are reduced by recombinant leptin in affected mice [14] and humans [15]. CLD is an extremely rare monogenic condition characterized by rapid weight gain in childhood mainly due to hyperphagia. Patients get morbid obesity, dyslipidemia, insulin resistance, glucose intolerance, and steatosis. The first clinical trials with leptin administration to subjects with CLD due to mutations in the leptin gene were reported in 1999. Treatment of these patients with leptin resulted in a profound weight loss with fat mass loss due to reduced appetite. Their dyslipidemia, insulin resistance, hyperglycaemia and immune function were markedly improved. Leptin replacement in CLD has shown to induce lipolysis indicated by an increase in circulating free fatty acids [16,17].

Leptin treatment has proven to be highly efficacious at reversing the deleterious metabolic derangements in individuals with CLD [18]. Recombinant methionyl human leptin (metreleptin) therapy is used together with diet to treat complications caused by leptin deficiency in people who have lipodystrophy. Metreleptin has been shown to reduce hyperglycaemia, dyslipidemia, and insulin resistance in patients with lipodystrophy syndromes. Metreleptin for congenital leptin deficiency has been in clinical use in the UK for over 15 years in an extended compassionate use programme [19,20]. Recent studies have highlighted the management of leptin replacement therapy in monogenic obesity, identifying two LEP variants with opposing functional effects. This groundbreaking research underscores the urgent need for a deeper understanding of the genetic factors contributing to obesity, paving the way for more personalized interventions [21]. In 1999, Dr. Farooqi’s research team published a seminal paper describing a nine-year-old girl with severe, early-onset obesity and undetectable serum leptin levels, who was treated with recombinant human leptin. The positive therapeutic response in this child with leptin deficiency established the crucial role of leptin in regulating body weight and appetite in humans [16]. Another key study examined the long-term effectiveness and safety of metreleptin in treating patients with partial lipodystrophy. The researchers found that metreleptin was well tolerated throughout the 14-year study period, with most adverse events being mild to moderate in severity. The most frequently reported side effects included abdominal pain, hypoglycaemia, and nausea [19].

Several mutations of *LEP* associated with CLD have been described in humans to date. These findings have been documented in detail elsewhere, often associated with high rates of consanguinity. Our group reported a novel homozygous missense mutation [NM_002303.3], c.350 G>T [p.C117F] in *LEP* associated with no serum leptin concentrations, hyperphagia, and early-onset obesity in two severely obese sisters from Colombia born from consanguineous parents [22]. In the present study, we evaluated the metabolic and endocrine effects of metreleptin administered to both aleptinemic sisters for one year as a leptin replacement therapy, included in a compassionate therapeutic programme specifically developed in Colombia on behalf of the sisters. One-year changes in body composition, energy intake, glycated haemoglobin (HbA1c), fasting plasma glucose, triglycerides, alanine and aspartate aminotransferases, and treatment-emergent adverse events (TEAEs) were measured.

## Methods

### Ethics statement, consent, and permissions

This study was conducted in accordance with the Declaration of Helsinki and approved by the Institutional Review Board (IRB) of Dexa Diab Servicios Medicos, Bogotá, Colombia. DEXA DIAB is a Colombian leading medical care and research centre authorized to perform national and international clinical research protocols (https://dexadiab.com). Informed written consent was obtained from both subjects. Both sisters also gave written consent to publish their identifying details and all efforts were carefully taken to anonymize both participants.

### Subjects

The only two leptin-deficient patients identified in Colombia to date were recruited for this study. They are part of a highly consanguineous family. The clinical manifestations and genetic diagnosis of these two cases have been reported previously [22]. Briefly, the two extremely obese sisters enrolled and previously referred to as OBX1 and OBX2 were identified while attending an endocrinology clinic due to early childhood-onset severe obesity. Clinical histories for each person were compiled, followed by a complete physical examination emphasizing the clinical characteristics observed. A cause of genetic origin was suspected. Pedigree information was compiled, and blood samples were taken for DNA sequencing chromatograms, laboratory tests, genetic and hormone analysis, and diagnostic images. OBX1 is currently a 27-year-old female. She was first seen in the practice setting at age 9 for early childhood-onset morbid obesity and severe chest acne. Progressive weight gain was observed reaching a BMI of 40 kg/m2 at 14 years old. Her BMI at baseline was 59 kg/m2. OBX2 is a 24-year-old female. She was first seen in the practice setting at age 6. She is the third child of the nuclear family and younger sister of OBX1. She consulted for early childhood-onset morbid obesity and primary amenorrhoea. Baseline physical examination revealed that her BMI was 60 kg/m2. Both patients had comorbidities such as hypertriglyceridaemia and needed additional treatments (lipid-lowering drugs) to overcome long-term cardiovascular clinical complications. The study was designed to focus on reversing excess of body fat, metabolic abnormalities and improving quality of life.

### Metreleptin administration

Patients were evaluated at baseline, biweekly for the first 4 months, and monthly from month 5 to 12. After completion of a baseline period to collect measurements and training, patients began 2.5 mg subcutaneous metreleptin once daily for the first month. Doses were titrated up to 5 mg daily during the course of treatment. We recommended daily evening administration to model the normal circadian variation in endogenous leptin which is characterized by a pulsatile circadian rhythm with marked nocturnal rise [23,24]. Metreleptin dose was adapted to tolerance and effectiveness, and data on anthropometric phenotypes, energy intake, lipid profile, glycated haemoglobin (HbA1c), haematologic, renal, and liver function tests were collected. Subcutaneously metreleptin was administered at 2.5 mg daily for one month (Table 1). We adhere to the standard adult dosage recommended for lipodystrophy treatment (2.5 mg subcutaneously once daily), with dosage adjustments made in 2.5 mg increments based on clinical response, up to a maximum of 5 mg. These adjustments were made based on clinical outcomes, including metabolic control, tolerability issues, or concerns regarding excessive weight loss. Additionally, we follow the safety guidelines established by the US FDA through a Risk Evaluation and Mitigation Strategy (REMS) for Metreleptin, which includes measures to ensure safe usage and an implementation system designed to support proper monitoring [24].

**Table 1.** Metabolic markers and anthropometric outcomes at baseline, 6- and 12-months in the patients included in the study (OBX1 age: 27 y.o.; OBX2 age: 24 y.o.

Phenotype	OBX1			OBX2		
Body weight, body composition and blood pressure (BP)	Pre/metreleptin	6th month	Final	Pre/metreleptin	6th month	Final
Weight (Kg)	133	108	85	131	113	105
Waist circumference (cm)	141	121	99	141	112	110
Fat mass (%)	60	53	46	58	56	52
BMI (kg/m2)	59	49	38	60	51	48
Systolic BP (mmHg)	110	109	110	150	100	96
Diastolic BP (mmHg)	84	68	73	81	61	53
Total food intake (kcal)	1387	1162	1016	1579	1344	1228
Metabolic, haematologic, renal and liver characteristics	110	109	110	150	100	96
Leptin (ng/mL)	0.0	20.7	3.2	0.0	4.1	0.5
HbA1C (%)	5.3	5.5	5.7	5.9	5.7	5.7
Glucose (mg/dl)	79	80	79	78	80	79
Insulin mlU/L	6.0	4.7	2.0	19.9	12.8	5.7
HOMA-IR	1.1	0.8	0.4	3.5	2.3	1.1
TyG Index	4.72	4.45	4.19	5.05	4.87	4.70
LDL cholesterol (mg/dl)	155	126	101	106	98	80
HDL cholesterol (mg/dl)	61	49	63	43	41	50
Triglycerides (mg/dl)	160	62	55	314	213	154
Total Cholesterol (mg/mL)	248	187	175	212	179	161
White Blood Cells (WBC) (10^3/uL)	5,720	5,440	4,120	8,140	6,715	6,210
Eosinophils	0.19 ×10^3/uL	0.11 ×10^3/uL	0.15 ×10^3/uL	0.13 ×10^3/uL	0.12 ×10^3/uL	0.10 ×10^3/uL
Creatinine (mg/mL)	0.65	0.5	0.61	0.5	0.5	0.52
Aspartate aminotransferase (U/I)	25	24	27	54	28	30
Alanine aminotransferase (U/I)	21	18	16	74	**29**	**29**

### Food intake and body composition

Patients were allowed to eat ad libitum so that the effects of Metreleptin on food intake and nutrient choice could be documented (Figures 2 and 3). Food records were obtained at regular intervals to assess dietary intake and analysed by using the DIAL software for assessing diets and food calculations (Madrid, Spain, 2013, http://www.alceingenieria.net/nutricion/descarga.htm). Body weight was recorded monthly. Body composition was measured by dual energy x-ray absorptiometry (DXA) scanning (GE Lunar Prodigy, GE Healthcare, USA).

### Biochemical, metabolic & safety analysis

At each monthly visit, adverse events (AEs) were reviewed. Fasting blood samples for clinical effects and safety measurements were collected monthly. Fasting plasma glucose (FPG) and lipid values were determined according to standard methods with the use of automated equipment (Beckman, Fullerton, CA). HbA1c (Vitros 5600, Ortho Clinical Diagnostics, USA), FPG, triglycerides, and a lipid-lipoprotein profile were gathered throughout the 12 month period (Figure 4). Safety endpoints included changes from baseline in systolic and diastolic blood pressure (mmHg), heart rate, electrocardiograms, white blood cells, creatinine, alanine aminotransferase (ALT), aspartate aminotransferase (AST) and the incidence of treatment-emergent AEs (TEAEs). Serum leptin levels were determined by immunoassays with the use of a commercial kit (Vitros 5600, Ortho Clinical Diagnostics, USA). Insulin was measured through the Immunoenzymometric assay. Triglyceride-glucose (TyG) index was gathered to determine insulin action impairment and calculated as TyG index = ln [Fasting triglyceride (mg/dl) × FPG (mg/dl)]/2, is a composite indicator composed of fasting triglyceride (TG) and FPG [25]. Subjects with an index of 4.49 or greater are likely to suffer from IR [26]. We also used the HOMA-IR (Homeostatic Model Assessment for Insulin Resistance) index to determine if insulin resistance was present in the sisters. A cut-off of 2.60 or higher is considered as a correlate of IR [27,28].

### Behavior

Both sisters received a validated instrument measuring general psychopathology, anxiety and depression using rating scales described in the Hamilton Depression instrument (HAM-D or HDRS). The HAM-D [29] is regarded as the ‘gold standard’ for assessing severity of depressive episodes in patients with mood disorders. Defined cut-off points and severity levels are as follows: > 23 = very severe; 19–22 = severe; 14–18 = moderate; 8–13 = mild; and < 7 = remission. The scale predominantly assesses cognitive and vegetative symptoms. Results are categorized as mild, moderate, or severe depression. This instrument was administered after a structured interview.

### Statistical analyses

All data is presented as absolute values. We calculated the differences from baseline to 12-month to understand the magnitude of impact from metreleptin on OBX1 and OBX2 phenotype. No a-prior or power calculations were required due to case series.

## Results

### Anthropometric markers and body composition

**Figure 1.**
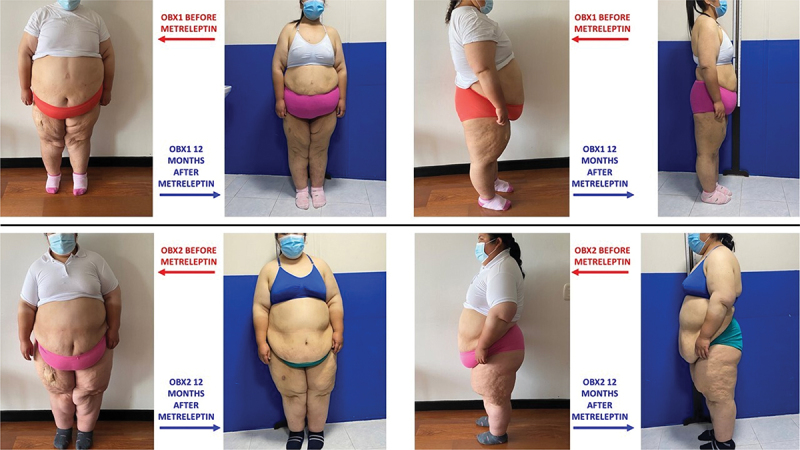
Colombian sisters before and 12 months after treatment.

Weight loss was observed within a week after the initiation of metreleptin. Patients’ BMI and fat mass dropped continuously throughout the study with significant change in their appearance (Figure 1). Table 1 describes their metabolic markers and anthropometric measurements during baseline, at 6 months and at the end of the 12-month period after administering metreleptin. The older sister (OBX1) had a baseline weight of 133 Kg, reaching 85 Kg (−36.1%) after 12 months (Δ = 48 Kg); her BMI went from 59 Kg/m2 to 38 Kg/m2 (Δ = 21); her % of body fat decreased with a Δ of 14%. OBX2 started a baseline weight with 131 Kg reaching 105 Kg (−19.9%) after 12 months (Δ = 26); her BMI went from 60 Kg/m2 to 48 Kg/m2 (Δ = 12); her % of body fat decreased with a Δ of 5% (Figure 3).

### Food intake

**Figure 2.**
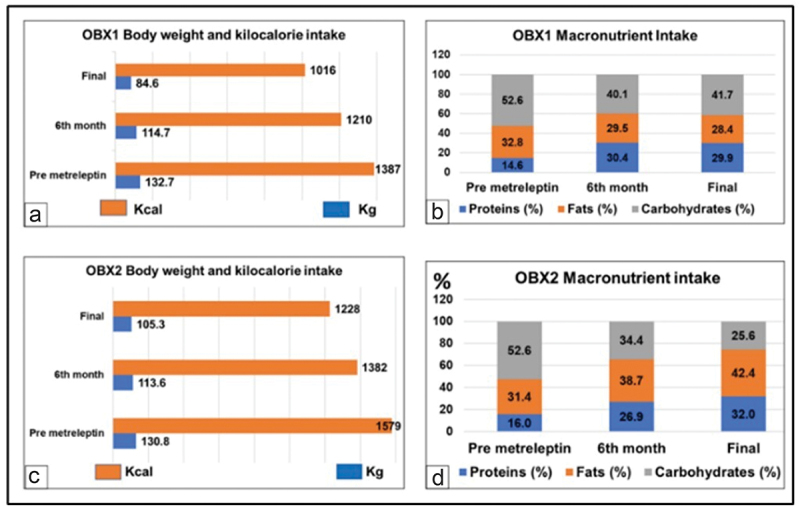
Body weight, macronutrient and kcal intake for OBX1 and OBX2.

**Figure 3.**
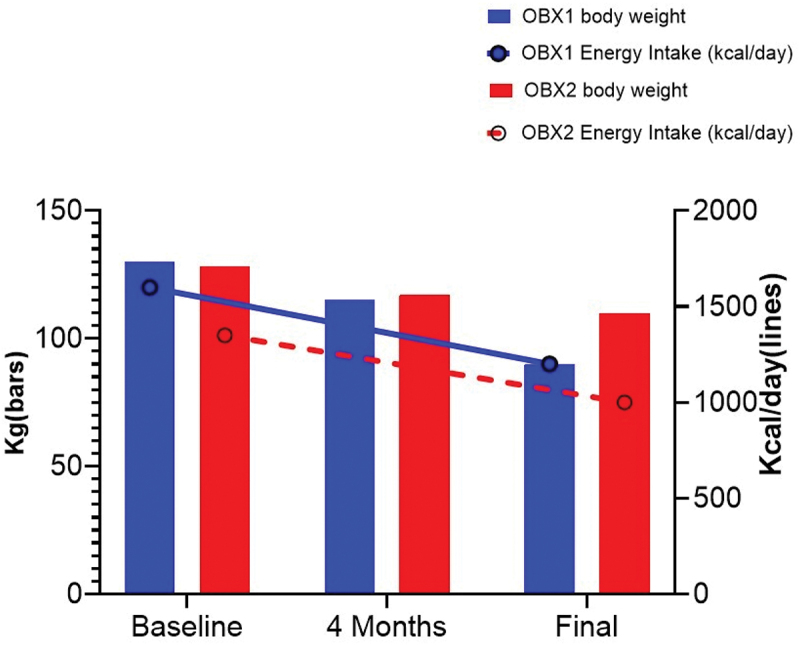
Weight loss and calorie intake trajectory at 6 and 12 months.

**Figure 4.**
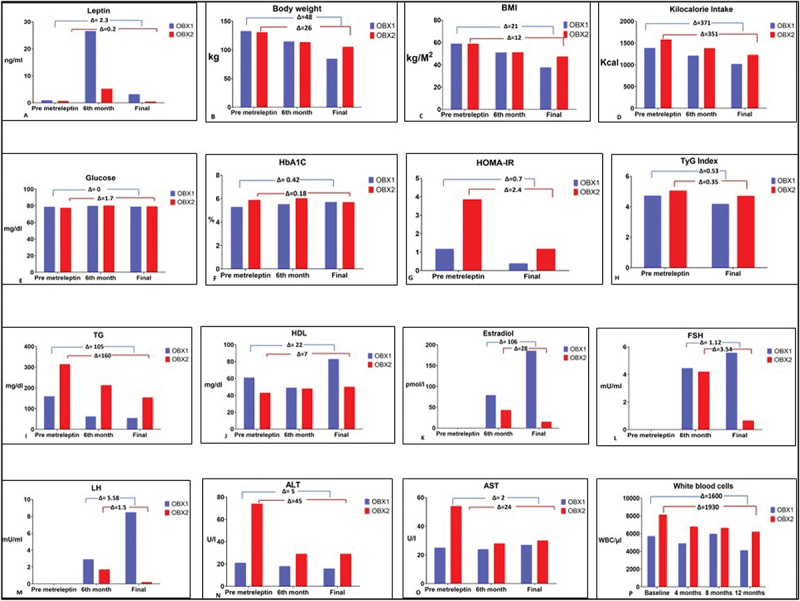
Baseline and 12-months delta (Δ) phenotypic differences before and after metreleptin between values to quantify overall changes in both OBX1 and OBX2 sisters.

Metreleptin therapy affected food consumption in both sisters. OBX1 had a kilocalorie intake of 1,387 Kcal before metreleptin administration. After 12 months of metreleptin, her kilocalorie intake was reported as 1,016 Kcal (Δ = 371 Kcal, Figures 2 (a), 3 and 4(d). Her premetreleptin macronutrient intake was 52.6% of carbohydrates (CHO), 32.8% fat, and 14.6% protein (prot) (Figure 2(b). OBX2 had an intake of 1,579 Kcal before metreleptin and 1,228 Kcal after 12-month metreleptin administration ((Δ = 351 Kcal, Figures 2(c), 3 and 4(d). Her premetreleptin macronutrient intake was 52.6% of carbohydrates (CHO), 31.4% fat, and 16.0% protein (prot) (Figure 2(d).

### Leptin levels, lipid and glucose metabolism

As shown in Table 1, leptin levels (ng/mL) for OBX1 were 0.0 at baseline, 20.7 after 6 months of metreleptin treatment, and 3.2 after 12 months. OBX2 exhibited leptin levels of 0.0 at baseline, 4.1 after 6 months, and 0.5 after 12 months of metreleptin therapy. FPG levels and HbA1c for OBX1 were 79 mg/dL and 5.3% at baseline, and 79 mg/dL and 5.7% after 12-month metreleptin. Her insulin levels were 6.0 mIU/L at baseline and 2.0 mIU/L after 12 months. Triglycerides were 160 mg/dL at baseline and 55 mg/dL after metreleptin. HDL-C levels were 61 mg/dL at baseline and 63 mg/dL 12 months later. OBX2 had FPG and HbA1c reported with 78 mg/dL and 5.9% at baseline, and 79 mg/dL and 5.7% after metreleptin. Her insulin levels were 19.9 mIU/L at baseline and 5.7 mIU/L after 12 months. Triglycerides were 314 mg/dL at baseline and 154 mg/dL after metreleptin. HDL-C levels were 43 mg/dL at baseline and 50 mg/dL after 12 months. These results offer novel perspectives into the effects of metreleptin in congenital leptin deficiency. The substantial reduction in BMI, along with improvements in circulating levels of leptin and metabolic parameters – particularly insulin sensitivity and lipid profiles – represent valuable contributions to the literature, especially within this rare genetic framework [8,30].

### Insulin resistance measurements through the HOMA-IR and triglyceride-glucose (TyG) index

Baseline for HOMA-IR in OBX1 was 1.1 and 0.4 after metreleptin therapy. OBX2 had a HOMA-IR of 3.5 and it decreased to 1.1 after metreleptin administration. The TyG index has been identified as a reliable alternative biomarker of insulin resistance (IR). IR is a state of decreased sensitivity and responsiveness to the action of insulin and has been identified as a hallmark of T2DM [31], even preceding diabetes for several years [32,33]. OBX1 had a baseline TyG index of 4.72 and 4.19 after 12 months under therapy. OBX2 had a baseline TyG index of 5.05, decreasing to 4.70 after metreleptin administration.

### Behavior

OBX1’s HAM-D results reported a cutoff point of 12 (mild) at baseline and a 6 (normal) after metreleptin. OBX2’s HAM-D results reported a cut-off point of 17 (mild) at baseline and a 2 (normal) after metreleptin.

## Discussion

Leptin replacement therapy is currently considered as the only effective treatment for monogenic leptin deficient forms of human obesity [34]. It has been documented that some individuals with an excess of body fat accompanied with undetectable or very low leptin levels, or with polymorphisms associated with lower leptin, might benefit from this therapy [18]. As noted in the results section, circulating leptin levels (ng/mL) for OBX1 and OBX2 varied from undetectable at baseline to elevated levels at 6 months, followed by a subsequent decline 12 months after metreleptin administration. Given the observed variability and fluctuations, we hypothesize that these changes may reflect individual responses to leptin replacement therapy. Further research is needed to identify potential factors contributing to this variability.

Leptin exerts dual effects on pancreatic β-cell function by suppressing insulin gene expression and secretion, and inhibiting ectopic lipid storage in islet cells, preventing lipotoxicity. Moreover, leptin has been proposed to protect the pancreatic islet beta-cells by acting on several pathophysiological steps involved in lipotoxicity and in glucagon production during diabetes, consequently preventing the accumulation of lipid intermediates in non-adipose tissue, therefore avoiding cellular dysfunction and death [35,36]. Metreleptin for injection, an analog of leptin, is the first FDA-approved therapy as an adjunct to diet to treat the complications of leptin deficiency in patients with congenital generalized or acquired generalized lipodystrophy in the USA. Metreleptin improves glycaemic metabolism in patients with both partial and generalized lipodystrophy, particularly those with undetectable leptin levels [25,26]. This improvement could be attributed to the reduction of lipotoxicity due to lipid overload, as indicated by changes in serum triglyceride and lipoprotein profiles, as well as decreased lipid accumulation in the liver and muscle tissues [37,38]. Additionally, leptin administration has been shown to increase adenosine monophosphate-activated protein kinase (AMPK) activity, leading to a significant reduction in triglyceride levels in both the liver and skeletal muscle. Therefore, metreleptin improves glucose metabolism and insulin sensitivity by upregulating AMPK activity in a way that differs from metformin [37,39].

The most evident phenotype clinically found in the Colombian sisters was extreme obesity [22,40]. OBX1 and OBX2 initial BMI was 59 and 60 kg/m2. After 12 months of treatment, they reached a BMI of 38 and 48 kg/m2 respectively. This was also the case for their weight and waist circumference (Table 1). Weight loss was achieved through a minimal instruction on dietary changes or on specific prescriptions to increase their physical activity. Most of the decrease in BMI after 12 months was attributed to fat mass loss, as measured by DXA (Figure 4) [41]. Both sisters had a lower kilocalorie intake as suggested by the nutritional assessment. Metreleptin therapy has been shown to reduce hunger and desire to eat in leptin-deficient humans, and significant weight loss is typical [18]. The drug has proven to be effective with an excellent safety profile [42,43]. This was the effect on food and macronutrient intake achieved in the Colombian sisters as shown in Figures 2 and 3. An interesting observation was that metreleptin changed the macronutrient content of both sisters’ food intake, with an increase in protein content and a substantial decrease in carbohydrate consumption. It was noticeable in both sisters that carbohydrates showed a greater reduction, with a striking 50% decrease in OBX2. We speculate that the caloric intake following metreleptin administration, perhaps indicated that what was relevant in the sister’s food intake was the change in macronutrient composition, not the impact on the total energy intake after metreleptin.

Regarding their lipid and glucose metabolism, before treatment, both sisters had normal FPG and HbA1c levels. OBX1 had normal insulin levels, HOMA-IR and TyG index. After 12 months these three key parameters of insulin-mediated glucose disposal improved. On the contrary, OBX2 had hyperinsulinemia and measurements of HOMA-IR and TyG index compatible with insulin resistance before treatment (Table 1 and Figure 4). After 12 months of metreleptin administration these parameters were within normal limits. One interesting observation was that their conventional measurements of their insulin-glucose axis (FPG and HbA1c) to diagnose type 2 diabetes were within normal limits under antidiabetic medication before metreleptin administration. It is important to keep in mind that these two sisters were frank diabetic before appropriate treatment for their extreme obesity and hyperglycaemia. Both sisters experienced full remission of diabetes, maintaining euglycemia and normal HbA1c levels without diabetes medications since the beginning of their 4th month under metreleptin administration. Leptin replacement also normalized their serum triglycerides and aminotransferases which may suggest improvement of non-alcoholic fatty liver disease (NAFLD). Metreleptin has been shown to enhance glucose metabolism and lipid profiles through several mechanisms: improving serum triglyceride and lipoprotein profiles, reducing VLDL and IDL cholesterol fractions, ameliorating impaired insulin sensitivity, regulating energy homoeostasis, insulin action, lipid metabolism, and immune function, lowering blood glucose levels, exerting anti-lipogenic effects, and acting both centrally and peripherally in the endocrine pancreas, liver, skeletal muscle, adipose tissue, immune cells, and cardiovascular system [44].

Before treatment, the sisters were diagnosed with hypogonadotropic hypogonadism through normal gonadotropin responses to GnRH stimulation as reported by their endocrinologist. After treatment, menstrual periods became regular in OBX1. OBX2 had primary amenorrhoea experiencing her first menstrual period after metreleptin. Recovery of menstruation evidenced that the hypogonadotropic hypogonadism was reversed, also evidencing a physiological pulsatility of the hypothalamic-pituitary-gonad axis. Although sex hormone and gonadotropin determinations were performed from baseline, 6 and 12 months, an increase in oestradiol levels can be seen in addition to the conclusive clinical fact of the recovery of cyclical menstruation. This would have been even more evident if such hormonal measurements had been obtained at certain times of their menstrual cycle.

**Figure 5.**
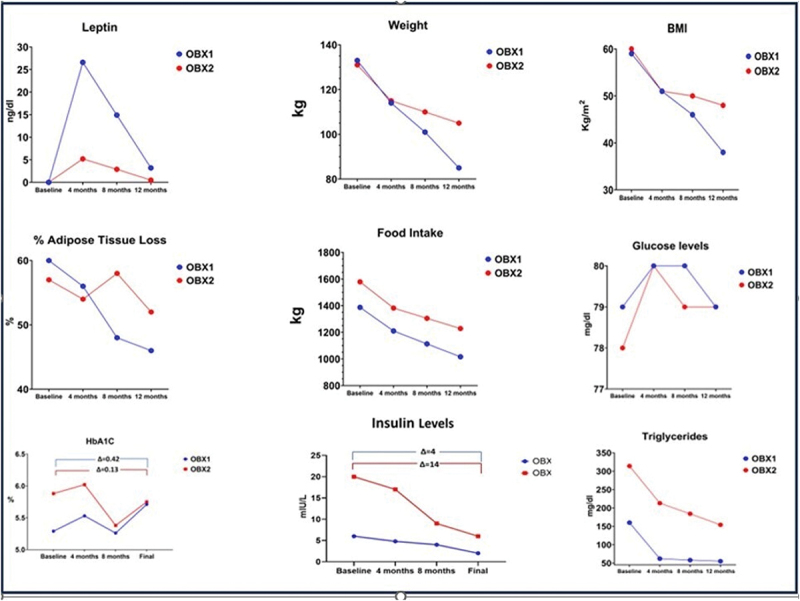
Leptin levels, weights, BMI, % of body fat, metabolic parameters of the insulin-glucose axis, and triglycerides.

As shown in Figure 5, the most interesting observation was the differences in response to the metreleptin treatment between OBX1 and OBX2. These different changes in time were present despite both sisters sharing the same environment and socioeconomics, the same household, and the same standard of treatment. OBX1 had a much better response to the 12-month leptin replacement than her younger sister. Her weight, fat% and BMI resulted in larger losses than OBX2. It would perhaps be quite difficult and speculative to understand or explain why OBX1 had higher leptin circulating levels during the time course of metreleptin administration. Both sisters started the 12-month treatment with normal levels of glucose and HbA1c under antidiabetic medication. However, OBX2 was hyperinsulinemic and insulin resistant. Their food intake clearly shows that both sisters improved their caloric intake and somehow managed to decrease their carbohydrate intake. These actions were more pronounced with OBX1. Nevertheless, there was a clear metabolic improvement in both sisters at the end of 12 months without diabetic medications. It is also evident an improvement in their quality of life and anxiety and depression levels as shown in the results from their HAM-D instrument before and after metreleptin. Personal communication with their endocrinologist revealed that they describe their lives after the treatment as ‘the best ever, given that they do not think in food all day anymore’.

Although metreleptin may have a contribution in decreasing food intake, it appears that the main action of metreleptin in correcting the metabolic abnormalities secondary to aleptinemia and lipotoxicity is based on the clearance of triglycerides from the muscle and the liver and the removal of ectopic fat from these and other tissues [30,38]. It has been described that rodent models lacking leptin action as a consequence of aleptinemia develop hyperphagia and obesity resulting in ectopic fat accumulation and steatosis in non-adipose tissues such as liver, heart, pancreatic islets, kidneys, and skeletal muscle [9,45]. Therefore, there is an excess of fatty acyl CoA entering nonoxidative metabolic pathways ultimately leading to lipotoxicity and lipoapoptosis [39,46]. It has been described that in patients with generalized lipodystrophy, long-term treatment with metreleptin resulted in sustained improvements by reversing the consequences of lipotoxicity: hypertriglyceridaemia, glycaemic control, and liver volume [47]. We found similar results in the sisters after 12 months of metreleptin administration (Figures 4 and 5).

Some potential limitations of this study should be pointed out. These include extremely small sample size, lack of controls, and some difficulty to gather reports of outcomes due to the complex integrative interactions with the governmental-base supportive staff and Colombian personnel regarding nutritional, pharmacological and motivational treatment adherence. Also, we did not measure if the sisters developed antimetreleptin antibodies. FDA-approved labelling for metreleptin includes a boxed warning on the risks of the development of antibodies with neutralizing activity resulting in increased risk of infection or worsening of metabolic control. However, both sisters showed normal levels of white blood counts and eosinophils during the 12 months of therapy (Table 1).

Despite these limitations, our results and findings in both sisters are consistent with most reports across different studies. As CLD is a very rare condition, the peer-reviewed publications regarding the use of metreleptin for CLD are very limited. However, the published evidence suggests that this treatment in CLD patients is safe and effective. As we showed in our results, it is quite clear that metreleptin administration exerts a positive effect on reduction of body weight and fat accumulation, also correcting the endocrine and gonadal abnormalities associated with CLD. One definitive achievement of this study was the fact to have been able to provide both aleptinemic sisters with a very expensive medication for 12 months despite the socioeconomic disadvantages existing in Latin America, including Colombia.

In conclusion, 1 year of metreleptin therapy resulted in marked body weight and fat mass loss, along with metabolic improvement, also reversing hyperphagia in both sisters with CLD. Improved leptin and insulin actions related to a decreased fat mass and distribution may have contributed to all medical benefits observed after metreleptin treatment. No safety concerns were observed throughout 1 year of treatment with metreleptin in both sisters. Nevertheless, the small sample size (only two subjects) and the absence of controls were significant limitations that impeded the generalization of results and broader implications of metreleptin administration over one year. This issue is closely tied to the rarity of the genetic mutation in the obese population. Additionally, further studies are needed to assess the long-term effects of metreleptin therapy and whether the metabolic improvements persist after treatment cessation. Long-term efficacy and the durability of metreleptin’s impact on metabolic variables have been reported after three years of treatment. This research showed that after this duration, mean values for A1C, FPG, triglycerides, and liver enzymes were lower than the recommended treatment targets for patients with elevated baseline levels of these variables [48]. Further investigation is needed to determine whether these effects can be sustained over time.

## Data Availability

Due to the nature of this research, the raw data supporting the conclusions of this article will be made available by the authors upon request.
